# A simple toolset to identify endogenous post-translational modifications for a target protein: a snapshot of the EGFR signaling pathway

**DOI:** 10.1042/BSR20170919

**Published:** 2017-08-02

**Authors:** Henrick Horita, Andy Law, Soonjin Hong, Kim Middleton

**Affiliations:** R&D Department, Cytoskeleton Inc., Denver, CO 80223, U.S.A.

**Keywords:** acetylation/deacetylation, epidermal growth factor receptor, post translational modification, phosphorylation/dephosphorylation, sumoylation, ubiquitins

## Abstract

Identification of a novel post-translational modification (PTM) for a target protein, defining its physiologic role and studying its potential cross-talk with other PTMs is a challenging process. A set of highly sensitive tools termed as Signal-Seeker kits was developed, which enables rapid and simple detection of PTMs on any target protein. The methodology for these tools utilizes affinity purification of modified proteins from a cell or tissue lysate, and immunoblot analysis. These tools utilize a single lysis system that is effective at identifying endogenous, dynamic PTM changes, as well as the potential cross-talk between PTMs. As a proof-of-concept experiment, the acetylation (Ac), tyrosine phosphorylation (pY), SUMOylation 2/3, and ubiquitination (Ub) profiles of the epidermal growth factor (EGF) receptor (EGFR)–Ras–c-Fos axis were examined in response to EGF stimulation. All ten previously identified PTMs of this signaling axis were confirmed using these tools, and it also identified Ac as a novel modification of c-Fos. This axis in the EGF/EGFR signaling pathway was chosen because it is a well-established signaling pathway with proteins localized in the membrane, cytoplasmic, and nuclear compartments that ranged in abundance from 4.18 × 10^8^ (EGFR) to 1.35 × 10^4^ (c-Fos) molecules per A431 cell. These tools enabled the identification of low abundance PTMs, such as c-Fos Ac, at 17 molecules per cell. These studies highlight how pervasive PTMs are, and how stimulants like EGF induce multiple PTM changes on downstream signaling axis. Identification of endogenous changes and potential cross-talk between multiple PTMs for a target protein or signaling axis will provide regulatory mechanistic insights to investigators.

## Introduction

The mammalian proteome has been estimated to contain multiple millions of unique proteoforms [1,2]. This level of complexity is derived from a relatively simple genome (approx. 25,000 genes), a transcriptome which increases the potential protein footprint to 100,000, and protein post-translational modifications (PTMs) which account for the vast increase in proteome complexity and an almost limitless potential for functional diversity [3–5]. For any given protein, a variety of PTM proteoforms offer a way to facilitate rapid cellular changes by altering the structure and function of the protein. Modifications include tyrosine phosphorylation (pY), ubiquitination (Ub), small ubiquitin-like modifier 2/3 (SUMOylation 2/3 (SUMO 2/3)), and acetylation (Ac), in addition to many others [6–9]. Specific proteoforms play a critical role in signal transduction, protein stability and turnover, protein–protein recognition and interaction, as well as spatial localization [10]. Importantly for human health and disease, misregulation of PTMs has been implicated in the progression of diseases like cancer, heart failure, neurologic, and metabolic diseases [11–15]; several emerging therapeutics targetting the Ac, Ub, and SUMOylation pathways serve to demonstrate the therapeutic potential of PTM targets [16–18].

Once thought to be mechanisms for subtle regulation of target proteins, the characterization of PTM profiles for proteins such as p53, epidermal growth factor (EGF) receptor (EGFR), protein kinase C, tubulin, τ, and histones have clearly demonstrated the central role for multiple, dynamic PTM proteoforms in regulating protein function and orchestrating cellular events [19–21]. Evidence from proteomic analysis suggests that greater than 70% of proteins are phosphorylated or ubiquitinated at some point [22], and the non-degradative roles of Ub in processes such as protein–protein interactions and signaling is now well established [23]. In many cases, PTMs have been shown to work in concert to orchestrate a specific protein function and recent studies have suggested that both co-operative and negative PTM cross-talk is a pervasive and fundamental cell regulatory mechanism [24–27]. Accordingly, there is significant interest in not only characterizing individual PTMs on a protein of interest (POI) but also in characterizing the temporal regulation and interplay of multiple PTMs on a given protein target and within a given signal transduction pathway.

Tools to examine endogenous PTM proteoforms in an unbiased manner are being developed in the MS-proteomics arena, including the peptide-based bottom-up approach and, in particular, a number of top-down MS-based methods in which whole protein targets are analyzed by MS [28–30]. While these approaches are generating exciting and insightful data regarding PTM proteoforms, there are currently several technical and biological challenges. Some of the technical challenges include protein abundance bias [31], protein size limitations (in top-down applications), and method sensitivity [2]. Often it is only a very limited pool of researchers that have studied any given POI, and therefore have the expertise and insight to know what experimental system, conditions, and timelines are necessary to study their POI. Their lack of mass spectrometric techniques/analytics expertise presents a significant barrier to examine proteoform function in their system/POI [32,33]. A set of tools that empower these researchers to simply and quickly look at any potential PTM without the need to develop specialized methods should greatly facilitate PTM discovery.

As a proof-of-concept, A431 cells were analyzed for Ac, pY, SUMO 2/3, and Ub PTM profiles of the well-studied EGFR–rat sarcoma (Ras)–c-Fos axis. This pathway was selected for several reasons: (i) the level of endogenous, non-EGF stimulated target proteins spans a range from abundant to low level expression (EGFR > Ras > c-Fos), which would give some indication of the dynamic range of the Signal-Seeker tools, (ii) our selected protein targets represent transmembrane (EGFR), cytoplasmic/membrane bound (Ras), and nuclear (c-Fos) proteins, which would give an indication as to the efficiency of Signal-Seeker tools to detect protein targets from a comprehensive range of cellular compartments, (iii) multiple reports of PTM proteoforms for this set of proteins are available in the literature [34–46]. These studies highlight the ubiquitious nature of PTMs, and how dynamically they change in response to physiologic stimulants like EGF. Having effective tools that can identify endogenous PTMs will aide in elucidating mechanistic regulation of a target protein or signaling pathway.

## Materials and methods

### Cell culture and reagents

A431 cells were grown in Dulbecco’s modified Eagle’s medium (DMEM) (A.T.C.C., VA) supplemented with 10% FBS (Atlas Biologicals, CO), and penicillin/streptomycin (ThermoFisher, MA). Trypsin/EDTA was obtained from Gibco (ThermoFisher, MA). Unless otherwise noted, chemicals were obtained from Sigma Chemical Co. (Sigma, MO). Recombinant EGF and c-Fos were obtained from Active Motif (Active Motif, CA). Recombinant Ras was obtained from Cytoskeleton, Inc. (Cytoskeleton, CO). Human EGF was obtained from Cytoskeleton, Inc. (Cytoskeleton, CO). For EGF stimulation experiments, A431 cells were serum restricted for 24 h with serum-free DMEM in order to synchronize the cells. The cells were then treated with 33 ng/ml EGF for 0.5, 2, 5, 15, and 60 min in individual 15-cm dishes (Corning, NY), followed by subsequent lysis with BlastR lysis buffer (Cytoskeleton, CO).

### Western blotting

A431 cells were lysed with ice-cold BlastR lysis buffer (Cytoskeleton, CO), radioimmunoprecipitation assay (RIPA), mPER (ThermoFisher, MA), immunoprecipitation (IP) lysis (ThermoFisher, MA), denaturing, and Laemmli lysis buffer containing a cocktail of N-Ethylmaleimide (NEM), trichostatin A (TSA), sodium orthovandate (Na_3_VO_4)_, and protease inhibitors (Cytoskeleton, CO). BlastR lysis buffer is a complete cell lysis reagent that comprises a proprietary mixture of detergents, salts, and other buffer additives. DNA was removed by passing the lysate through the compressible BlastR filter system (patent pending, Cytoskeleton, CO). After dilution with BlastR dilution buffer, protein concentrations were determined with Precision Red Advanced protein reagent (Cytoskeleton, CO), and measured at 600 nm OD. Protein lysate samples were separated using Tris-glycine SDS/PAGE (ThermoFisher, MA) and transferred to Immobilon- P membranes, polyvinylidene fluoride (PVDF) (Millipore, MA). Membranes were blocked for 30 min at room temperature (RT) in Tris-buffered saline (10 mM Tris/HCl, pH 8.0, 150 mM NaCl) containing 0.05% Tween-20 (TTBS) and 5% milk (Thrive Life, UT), and then incubated with 0–2.5% milk in TTBS solution containing primary antibodies for 1–3 h at RT. Membranes were washed in TTBS for 3× for 10 min, prior to secondary antibody for 1 h at RT. Bound antibodies were visualized with horseradish peroxidase-coupled secondary antibodies and chemiluminescent reagent (Cytoskeleton, CO) according to the manufacturer’s directions. Antibodies used: EGFR (Millipore, MA), Ras (Cytoskeleton, CO), c-Fos (Abcam, MA), SUMO 2/3-HRP (Cytoskeleton, CO), tubulin (Cytoskeleton, CO), Flotillin-2 (Abcam, MA), E-cadherin (Abcam, MA), HSP90 (Abcam, MA), hexokinase 1 (Abcam, MA), AIF (Abcam, MA), histone H3 (Abcam, MA), cJUN (ThermoFisher, MA), p21 (Abcam, MA), HRP-anti-mouse secondary (Cytoskeleton, CO), HRP-anti-sheep secondary (Cytoskeleton, CO), and HRP-anti-rabbit secondary (Jackson ImmunoResearch, PA). Changes were quantitated by densitometry using ImageJ software (rsb.info.nih.gov).

### IP assay

A431 cells were lysed with ice-cold BlastR lysis buffer containing a cocktail of NEM, TSA, Na_3_VO_4_, and protease inhibitors (Cytoskeleton, CO). DNA was removed by passing the lysate through the compressible BlastR filter system (patent pending, Cytoskeleton, CO). After dilution with BlastR dilution buffer, protein concentrations were determined with Precision Red Advanced Protein Reagent (Cytoskeleton, CO), and measured at 600 nm OD. Samples were immunoprecipitated, using Signal-Seeker kits, with equal protein concentration and IP volumes according to the manufacturer’s protocol (Cytoskeleton, CO). The appropriate amount of pY beads (APY03-beads), Ub beads (UBA01-beads), SUMO 2/3 beads (ASM24-beads), Ac beads (AAC01-beads or 15E12-beads), IgG beads (CIG01), or Ub control beads (CUB01) were added to the respective samples for 1–2 h and rotated at 4°C. After incubation, the affinity beads from each sample were pelleted and washed 3× with BlastR wash buffer. Bound proteins were eluted using bead elution buffer (Cytoskeleton, CO) and detected by Western immunoblotting. For reciprocal EGFR IP experiment, samples were incubated with 8 μg of EGFR antibody (Millipore, MA) for 1–2 h at 4°C on an end-over-end tumbler. Fifty microliters of 50% slurry of Protein G beads (Biovision, CA) was added to each sample and incubated for 2 h rotating at 4°C. After incubation, the resin from each sample was pelleted and washed 3× with BlastR wash buffer. Bound proteins were eluted using bead elution buffer and detected by Western immunoblotting.

## Results

### Detection of multiple PTMs with a single assay buffer

**Figure 1:**
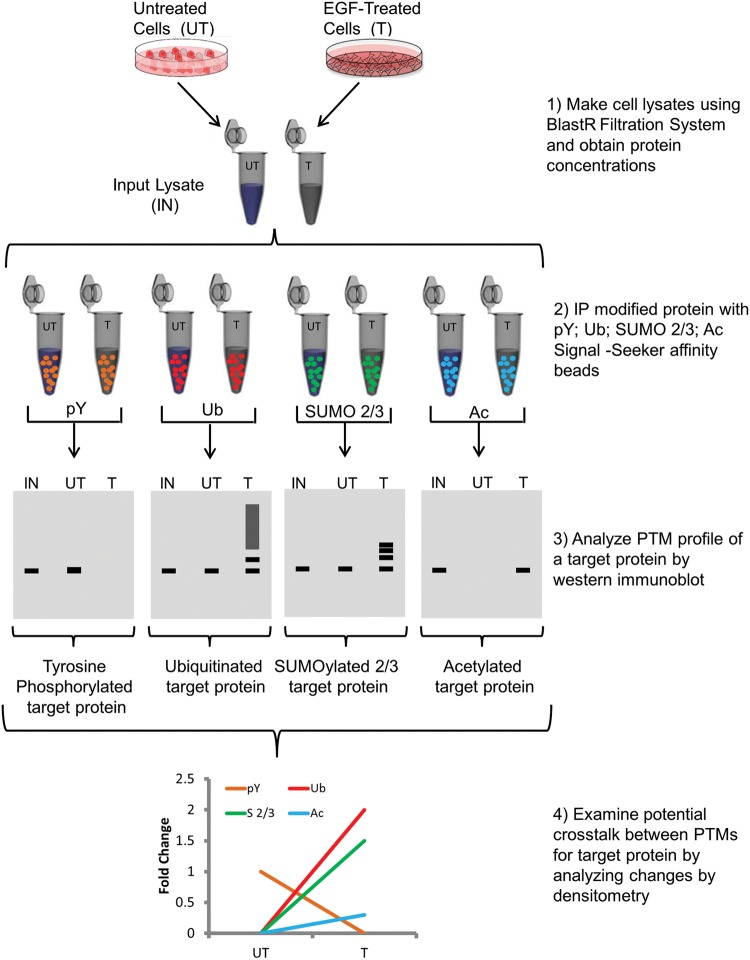
Workflow of Signal-Seeker PTM identification kits Diagram depicting steps performed in order to obtain pY, Ub, Sumo 2/3, and Ac PTM profiles for a POI.

**Figure 2:**
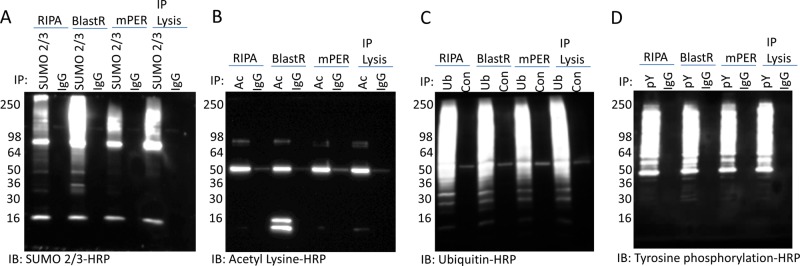
Comparison of BlastR lysis buffer with non-denaturing lysis buffers A431 cell lysate made with RIPA, BlastR, mPER, or IP lysis was immunoprecipitated with (**A**) SUMOylated 2/3 affinity beads or control beads, (**B**) acetyl lysine affinity beads or control beads, (**C**) Ub affinity beads or control beads, (**D**) phosphotyrosine affinity beads or control beads. Total SUMOylation 2/3, Ac, Ub, and tyrosyl phosphorylation profiles were detected with their respective antibodies.

Identification of a novel PTM for a target protein, defining its physiologic role, and studying its potential cross-talk with other PTMs is still a challenging process. In order to effectively analyze pY, Ub, SUMO 2/3, and Ac PTMs on any POI, robust affinity reagents (Signal-Seeker kits), and a unique lysis system was developed. Investigating all four PTMs in the same lysis system is essential because it allows users to gain a better picture of potential PTM cross-talk. Figure 1 shows a diagram of the workflow with cell lysis occurring at step 1; consequently, if each PTM were studied in their own buffer system, it would increase the time and resources needed to obtain the same experimental results. Identifying a single lysis system that would enable optimal enrichment of all four PTMs was problematic, because SUMOylation in particular was primarily studied using a strong denaturing buffer relative to the other PTMs [47]. Conversely, strong denaturing buffers may disrupt the integrity of some affinity reagents used to study modifications like Ub [48]. BlastR buffer, a denaturing lysis buffer, was developed to effectively isolate proteins from all cellular compartments (Supplementary Figure S1), while also enabling effective (IP) of these four PTMs. Utilizing the BlastR lysis buffer allowed for isolation of pY-, Ub-, SUMO 2/3-, and Ac-modified proteins, unlike RIPA and other non-denaturing buffers that showed incomplete profiles of SUMO 2/3- and Ac-modified proteins (Figure 2).

### Rapid detection of the four PTMs for EGFR

**Figure 3:**
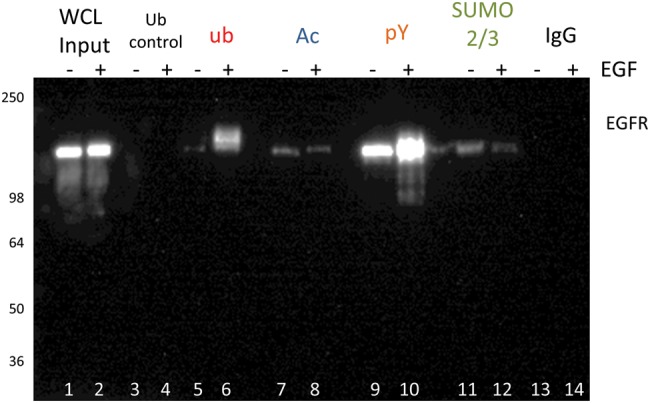
Rapid detection of the four PTMs for EGFR Serum-restricted A431 cells were either unstimulated or stimulated with EGF for 5 min prior to lysis with BlastR lysis buffer. Whole cell lysate (WCL) was analyzed for EGFR levels (lanes 1,2). Ubiquitin control beads (CUB02) were used to immunoprecipitate non-specific binding to ubiquitin beads, and serves as a control for UBA01-beads (lanes 3,4). Ubiquitin-binding beads (UBA01) were used to immunoprecipitate ubiquitinated proteins (lanes 5,6). Acetyl lysine-binding beads (15E12) were used to immunoprecipitate acetylated proteins (lanes 7,8). Phosphotyrosine binding beads (APY03) were used to immunoprecipitate tyrosine-phosphorylated proteins (lanes 9,10). SUMO 2/3 binding beads (ASM24) were used to immunoprecipitate SUMOylated 2/3 proteins (lanes 11,12). IgG binding control beads were used to immunoprecipitate non-specific binding proteins, and serves as a control for the antibody-based affinity beads: APY03-beads, ASM24-beads, and 15E12 beads (lanes 13,14). All samples were separated by SDS/PAGE and analyzed by Western immunoblotting using an EGFR antibody to identify changes in EGFR PTMs in response to EGF. Shown is a representative Western blot from *n* ≥3 independent experiments.

As an example of the ability of the Signal-Seeker kits to analyze all four PTMS for any POI, untreated and EGF-stimulated A431 cells were lysed with BlastR buffer and pY, SUMO 2/3, Ub, and Ac-modified EGFR PTMs were captured with Signal-Seeker affinity beads or control beads. Importantly, ubiquitin affinity beads (UBA01) utilized its own control beads (CUB02), because it is based on ubiquitin-binding domain (UBD) technology; thus, the control beads are derived from mutated UBDs (that do not bind ubiquitin) conjugated to the bead matrix. Alternatively, pY, SUMO 2/3, and Ac affinity beads are all antibody-based affinity matrices; therefore, they all use the same IgG control beads to identify non-specific interactions. Enriched pY, SUMO 2/3, Ub, Ac, and control beads samples were separated by SDS/PAGE, transferred on to PVDF, and analyzed by immunoblotting with an EGFR antibody (Figure 3). EGFR in particular has a well-characterized PTM profile, and previous publications have shown that EGFR can be modified by all the four PTMs [34–39]. The findings in Figure 3 confirmed that EGFR is tyrosine phosphorylated, SUMOylated 2/3, ubiquitinated, and acetylated. This is the first report in which all four PTMs of EGFR have been analyzed simultaneously in a single lysate system.

### Validate EGFR SUMO 2/3 modification

**Figure 4:**
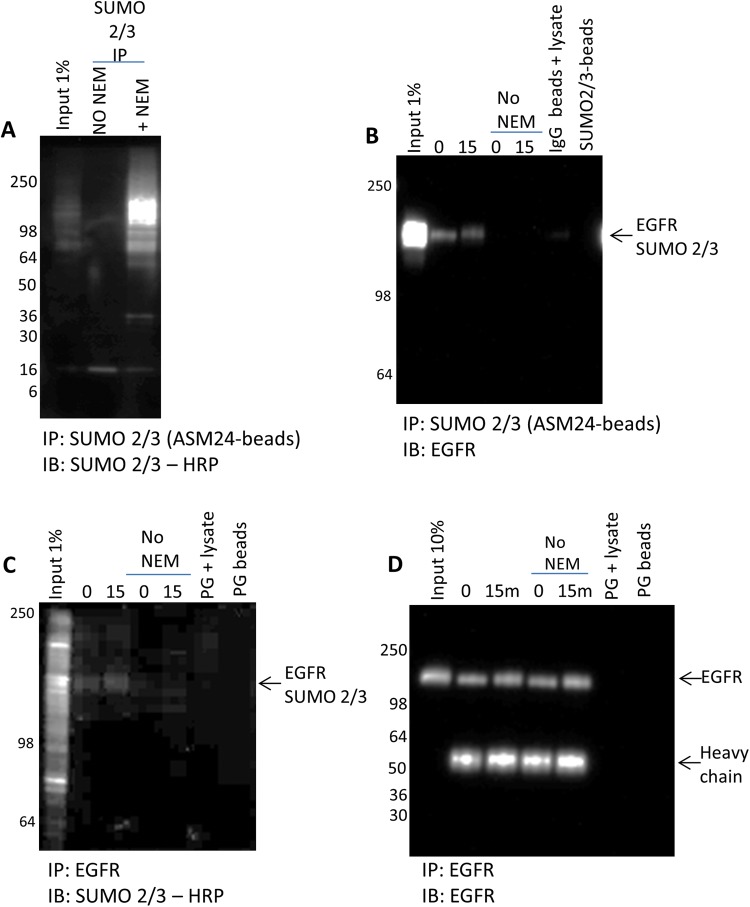
Validate EGFR SUMO 2/3 modification (**A**) A431 cells were harvested with BlastR lysis buffer with or without NEM. Lysates were incubated with ASM24 beads. Samples were separated by SDS/PAGE and analyzed by Western blot for total SUMOylated proteins with SUMOylated 2/3-HRP antibody. (**B**) Untreated and 15 min EGF-treated lysates were incubated with ASM24 beads. IgG control beads were incubated with untreated A431 lysate with NEM to identify non-specific binding. Samples were separated by SDS/PAGE and analyzed by Western blot for SUMOylated 2/3 EGFR with an EGFR antibody. (**C**) Untreated and 15 min EGF-treated lysates with or without NEM were incubated with EGFR antibody and protein G beads. Protein G beads alone were added to untreated A431 lysate with NEM to identify non-specific bead binding. Samples were separated by SDS/PAGE and analyzed by Western blot for SUMOylated 2/3 EGFR with sumoylated 2/3-HRP antibody. (**D**) Untreated and 15 min EGF-treated lysates with or without NEM were incubated with EGFR antibody and protein G beads. Protein G beads alone were added to untreated A431 lysate with NEM to identify non-specific bead binding. Samples were separated by SDS/PAGE and analyzed by Western blot for total EGFR with an EGFR antibody.

Identification of EGFR SUMO 2/3 was reported previously using a proximity ligation assay; however, as the focus of that study was on EGFR SUMO-1 modification, the findings on EGFR SUMO 2/3 were not pursued beyond that preliminary identification [39]. Thus, while data shown here (Figure 3) were complementary to those in the previous findings, further confirmation that EGFR was SUMO 2/3 modified was warranted [39]. Two approaches were taken to further confirm that EGFR was SUMO 2/3 modified. First, the IP of SUMO 2/3 was performed with or without the de-SUMOylase inhibitor, NEM, in the lysis buffer to confirm EGFR’s SUMO 2/3 status. Removing NEM from the lysis buffer significantly decreased the number of proteins that were SUMOylated, as determined by an overall decrease in the immunoprecipitated SUMO 2/3 profile (Figure 4A). Without NEM in the lysis buffer, the EGFR proteins were not captured and identified using SUMO 2/3 affinity beads, presumably because the EGFR proteins were de-SUMOylated (Figure 4B). To further validate the SUMO 2/3 EGFR finding, the reciprocal IP using an EGFR antibody was performed (Figure 4C,D). SUMO 2/3 of EGFR was examined in the present study using a SUMO 2/3-HRP antibody. The results confirmed that EGFR was SUMO 2/3 modified, and this modification of EGFR was diminished in the absence of NEM (Figure 4C). Figure 4D shows the total EGFR immunoprecipitated using an EGFR antibody; importantly, isolation of total EGFR is similar to with or without NEM, suggesting that the loss of the SUMO 2/3 EGFR-modified signal in Figure 4C is not due to inefficient IP in the samples without NEM. Of note, the quality of the data from Figure 4B compared with C highlights the benefit of working with IP-competent PTM targetting affinity beads compared with having to optimize a protein-specific antibody to perform the IP.

### Detect dynamic changes in all four PTMs for EGFR to identify potential PTM cross-talk

**Figure 5:**
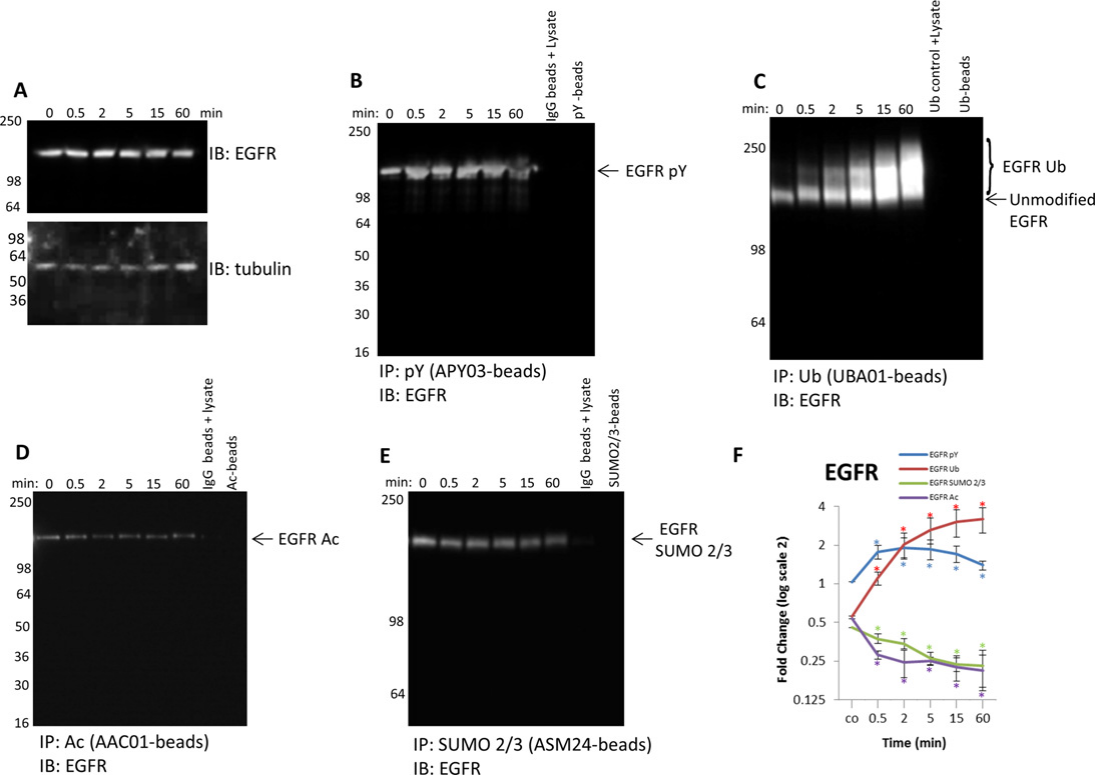
Detect endogenous, dynamic changes of the four PTMs for EGFR (**A**) Serum-restricted A431 cells were stimulated with EGF for the given time period. Whole cell lysate (WCL) was analyzed for EGFR levels. Tubulin was used as a loading control. Unstimulated and EGF-treated A431 lysates were incubated with (**B**) APY03-beads or IgG control beads to immunoprecipitate tyrosine-phosphorylated proteins and analyzed for tyrosine phosphorylated EGFR, (**C**) UBA01-beads or CUB02 control beads to capture ubiquitinated proteins and analyzed for ubiquitinated EGFR, (**D**) acetyl lysine binding beads or IgG control beads to immunoprecipitate acetylated proteins and analyzed for acetylated EGFR, (**E**) and ASM24-beads or IgG control beads to immunoprecipitate SUMOylated 2/3 proteins and analyzed for SUMOylated 2/3 EGFR. Shown are representative Western blots from *n*≥3 independent experiments. (**F**) Quantitation of densitometric analysis of EGFR PTMs. Error bars represent S.E.M. *t* test statistical analysis was performed. *P<0.05.

The Signal-Seeker kits are well suited to investigate potential cross-talk, because analyses of all PTMs for any given POI are performed with endogenous protein from a single lysate. To highlight this attribute, pY, SUMO 2/3, Ub, and Ac of EGFR were examined over a timecourse of EGF stimulation. Autophosphorylation of EGFR tyrosine residues occurs in response to EGF stimulation and receptor dimerization, and this PTM modification is necessary for recruitment and activation of downstream targets in the EGF/EGFR pathway [34,49]. Signal-Seeker pY affinity beads efficiently captured tyrosine-phosphorylated EGFR, and a dynamic change in the population of tyrosine-phosphorylated EGFR, but not total EGFR, was observed when examined over the EGF timecourse (Figure 5A,B). The EGFR protein is also ubiquitinated in response to EGF stimulation as a regulatory mechanism to suppress EGF signaling [35,36], and data obtained with Signal-Seeker Ub affinity beads corroborated these previous findings (Figure 5C). Work by Goh et al. [38] first identified EGFR Ac, and determined that it may play a role in EGFR internalization, but the study provided no information about dynamic regulation of EGFR Ac by EGF. Signal-Seeker acetyl lysine affinity beads were used to capture acetylated EGFR and showed that EGFR Ac rapidly decreased in response to EGF, and this decrease was maintained even at 1 h (Figure 5D). Investigation of less well-studied modifications like SUMOylation have also been performed on EGFR, and while there is convincing evidence that EGFR is SUMO-1 modified, the characterization of SUMO 2/3 has not been well-defined [39]. The data showed that EGFR is SUMO 2/3 modified; furthermore, the modified EGFR SUMO 2/3 proteoform is decreased in response to EGF stimulation (Figure 5E).

These findings highlighted that EGFR is modified by all the four PTMs in response to EGF stimulation, but the specific dynamics are different for each PTM (Figure 5F). Importantly, the densitometric data showed a rapid enhancement of tyrosine-phosphorylated EGFR that tapered off at 1 h. The decrease in tyrosine-phosphorylated EGFR occurred simultaneously with an increase in EGFR Ub, alluding to a potential cross-talk between these two PTMs. Previous reports have shown that Ub of EGFR down-regulates tyrosine-phosphorylated EGFR; thus establishing a link between pY and Ub of EGFR [35]. Further examination of the densitometric data showed a decrease in both EGFR SUMO 2/3 and Ac that was inverse to EGFR pY, possibly indicating potential cross-talk with these modifications as well. Having the ability to track endogenous changes in multiple PTMs for a target protein will shed light on the potential cross-talk between regulatory PTM modifications.

### Characterize target PTMs for EGFR–Ras-c–Fos axis: focus on Ub and SUMO 2/3

**Figure 6:**
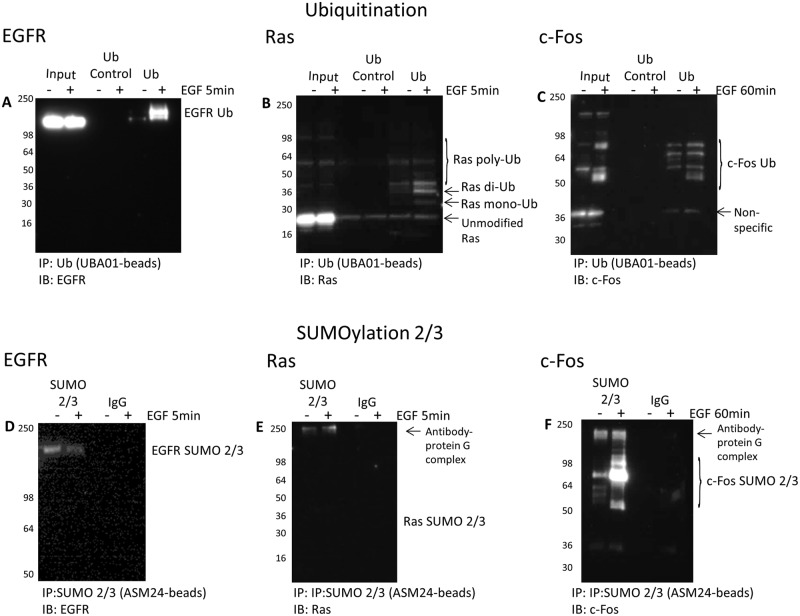
Characterize Ub and SUMO 2/3 for the EGFR–Ras-c–Fos axis Serum-restricted A431 cells were either unstimulated or stimulated with EGF for 5 or 60 min prior to lysis with BlastR lysis buffer. (**A**–**C**) Samples were immunoprecipitated with ubiquitin control beads (CUB02) or ubiquitin-binding beads (UBA01). Samples were separated by SDS/PAGE and analyzed by Western blot for (A) EGFR, (B) Ras, and (C) c-Fos to identify the ubiquitinated species for these proteins in the EGFR signaling pathway. Shown are representative Western blots from *n*≥3 independent experiments. (**D**–**F**) Samples were immunoprecipitated with IgG control beads or SUMO 2/3 binding beads (ASM24). Samples were separated by SDS/PAGE and analyzed by Western blot for (D) EGFR, (E) Ras, and (F) c-Fos to identify the SUMOylated 2/3 species for these proteins in the EGFR signaling pathway. Shown are representative Western blots from *n*≥3 independent experiments.

EGF stimulation is known to activate multiple kinase signaling pathways downstream of EGFR. However, we wanted to determine if the EGFR–Ras-c–Fos axis also undergoes dynamic Ub or SUMO 2/3 changes in response to EGF stimulation. The Ub profile of three key proteins in the EGF/EGFR signaling pathway was investigated to highlight the utility of the Signal-Seeker tools to identify Ub modifications of multiple target proteins. Changes in EGFR Ub, Ras Ub, and c-Fos Ub in response to EGF was effectively identified (Figure 6A–C). Importantly, an endogenous mono- and di-Ub signal of Ras (Figure 6B) was observed, and was similar to previously published results with transfected H-Ras [42]. Ubiquitinated c-Fos was observed in both unstimulated and EGF-treated samples, but a distinct pattern of c-Fos Ub was discernable with EGF treatment (Figure 6C).

The SUMO 2/3 profile for these three proteins in the EGF/EGFR signaling axis was also obtained (Figure 6D–F). There was no evidence for SUMO 2/3 modification of Ras in either unstimulated or EGF-treated conditions, which aligns with the lack of supporting data in the literature (Figure 6E). Unlike Ras, c-Fos was significantly SUMO 2/3 modified in response to EGF treatment (Figure 6F), and these data are similar to previously published findings using serum stimulation, which showed that c-Fos SUMO 2/3 modification altered its transcriptional activity [43]. The Ub and SUMO 2/3 data for these three target proteins are summarized in Table 1. Additional information regarding the pY and Ac PTM profile for these three target proteins are also included in the table and in Supplementary data (Supplementary Figures S2 and S3). Collectively, these data highlight how highly modified this signaling axis is, and the potential PTM cross-talk that occurs during physiologic stimulation with EGF.

**Table 1:** Summary of EGFR, Ras, and c-Fos PTM profile

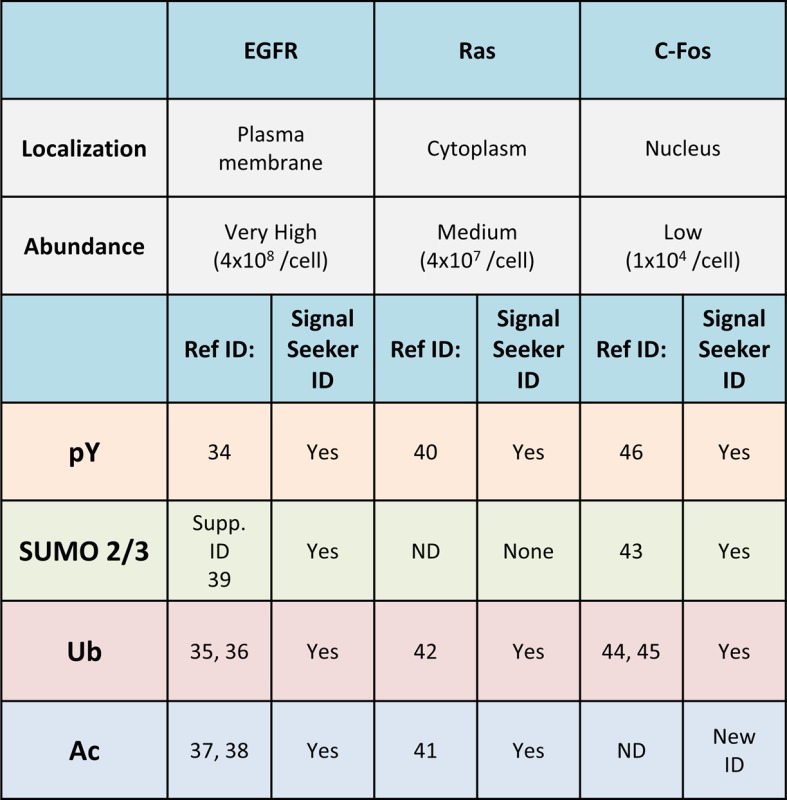

### Investigating endogenous Ras Ub is critical for regulatory insight

**Figure 7:**
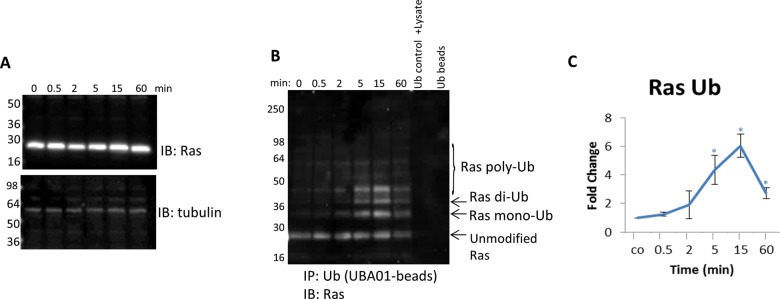
Detect endogenous, temporal changes of Ras Ub in response to EGF stimulation (**A**) Serum-restricted A431 cells were stimulated with EGF for the given time period. Whole cell lysate (WCL) was analyzed for Ras levels. Tubulin was used as a loading control. (**B**) Unstimulated and EGF-treated A431 lysates were incubated with ubiquitin-binding beads (UBA01) to immunoprecipitate ubiquitinated proteins or ubiquitin control beads (CUB02). Samples were separated by SDS/PAGE and analyzed by Western immunoblotting using a pan Ras antibody to identify ubiquitinated pan Ras. Shown are representative Western blots from *n*≥3 independent experiments. (**C**) Quantitation of densitometric analysis of endogenous ubiquitinated Ras in response to EGF stimulation. Error bars represent S.E.M. *t* test statistical analysis was performed. *P<0.05.

Di-Ub of Ras was shown to be important for regulating Ras activation [42]; thus, understanding how this PTM is regulated physiologically is critically important. The data in Figure 6B showed that the endogenous di-Ub of Ras was dynamic and significantly up-regulated in response to EGF stimulation, which was contrary to the Jura et al. [42] finding that showed a constitutive di-Ub of H-Ras using an overexpression system. To investigate the temporal nature of endogenous Ras di-Ub, a timecourse with EGF stimulation was performed. Figure 7A showed no significant change in total Ras protein in response to EGF. Conversely, a dynamic and significant increase in Ras di-Ub as early as 5 min was observed (Figure 7B,C). There was a peak in di-ubiquitinated Ras at 15 min that was sustained above basal conditions even after 1 h of EGF treatment (Figure 7B,C). These data highlight the benefit of having tools that effectively capture endogenous, physiologic changes in a target protein’s PTM profile.

### Validate novel c-Fos Ac modification

**Figure 8:**
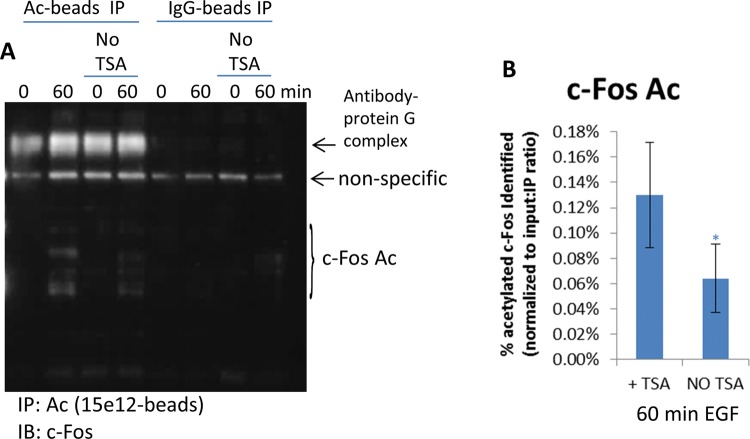
Identify and validate c-Fos Ac (**A**) Untreated or 60 min EGF-treated A431 cells were harvested with BlastR lysis buffer with or without TSA. Lysates were incubated with acetyl lysine binding beads or IgG control binding beads. Samples were separated by SDS/PAGE and analyzed by Western blot for acetylated c-Fos with a c-Fos antibody; shown are representative Western blots from *n*≥3 independent experiments. (**B**) Quantitation of densitometric analysis of c-Fos Ac from lysates with or without TSA. Samples were normalized to total c-Fos, as well as, input: IP ratio (0.008). Error bars represent S.E.M. *t* test statistical analysis was performed. *P<0.05.

Investigation of the PTMs of c-Fos resulted in the identification of pY, SUMO 2/3, and Ub c-Fos (Supplementary Figure S3), which has been reported previously [43–46]. Additionally, Supplementary Figure S3 showed that c-Fos was also acetylated at 1 h. To further confirm that c-Fos was acetylated, the IP experiment was performed in the absence of TSA, which resulted in a 50% decrease in c-Fos Ac (Figure 8A,B). The acetylated c-Fos represented only 0.13% (Figure 8B) of the total c-Fos population which was approx. 13500 c-Fos molecules per A431 cell (Supplementary Figure S4). Collectively, these data infer that the Signal-Seeker kits can detect endogenous PTMs as low as 17 molecules per cell.

## Discussion

### Effective identification of novel PTM proteoforms and potential regulatory mechanisms

In the present study, the Signal-Seeker PTM detection system was used to identify pY, Ub, SUMO 2/3, and Ac profiles for three target proteins in the EGF/EGFR signaling pathway, which resulted in the confirmation of ten previously identified proteoforms as well as identification of c-Fos Ac. Conversely, SUMO 2/3 modification of Ras was not detected, which corresponds with zero publications or reports on this proteoform in the literature. It is important to note that negative detection of a PTM for a target protein can be due to a multitude of reasons, and does not definitively prove that the protein is incapable of being modified by that particular PTM. For example, a particular target protein may only be modified under specific conditions, which may not have been examined in the present study. Additionally, PTMs may be cell-type specific, extremely low in abundance or affinity for a particular affinity reagent, which may influence isolation. Another point of potential false positive or false negative data are protein–protein interactions that are not disrupted by the lysis buffer. While the possibility of protein–protein masking is diminished in the BlastR system due to its denaturing capabilities; extremely strong protein interactions may not be disrupted even under these conditions. Additionally, the fact that dilution of the lysate occurs prior to IP allows for the possibility of re-association of proteins. Ongoing studies are being performed to assess the detection limit.

These findings demonstrate the fidelity of the data produced by the Signal-Seeker kits relative to previous PTM identifications in the literature, and suggests that it may be an effective tool to systematically identify the PTM profile of any protein or pathway of interest. Beyond identification of novel PTMs, the Signal-Seeker kits identified temporal changes to several of the PTMs in response to EGF, which may be regulatory on/off mechanisms. These studies provided preliminary information that two or more PTMs may be working co-operatively or in opposition (Figure 5 and Supplementary Figure S2), and provide a rational for futher investigation of these PTMs in combination. Ultimately, this system provides a simple and effective method to investigate one or more PTMs of several target proteins, and is complementary to the existing tools used to study PTMs.

### Universal lysis system maximizes PTM identification from all cellular compartments

Utilizing a standardized, non-denaturing buffer system, like those normally used to study pY and Ub PTMs [48], may lead to inefficient isolation of proteins from nuclear and membrane cellular fractions (Supplementary Figure S1) potentially leading to incomplete datasets. The present study describes a denaturing lysis system that was developed to study pY, Ub, SUMO 2/3, and Ac PTMs of a protein in the same lysate, which optimizes the time and resources required to determine if a specific POI is modified by these four PTMs. Importantly, the protein profile isolated with the BlastR lysis system was superior to RIPA buffer and other buffers (mPER, IP lysis) commonly used in IP asays; in particular, BlastR buffer was more efficient at isolating membrane and nuclear proteins (Supplementary Figure S1). BlastR buffer was comparable with cell lysis with Laemmli buffer, and sufficiently isolated proteins from membrane, nuclear, mitochondrial, and cytoplasmic compartments (Supplementary Figure S1). Unlike Laemmli buffer, the BlastR system allowed for easy protein quantitation with conventional protein assays, and maintained robust IP capability and PTM detection, which are all important for measuring changes in a target protein’s PTM profile. The system also utilizes a specialized filter to effectively remove genomic DNA contamination which can significantly interfere with IP and Western blot assays, and is a common contaminant in denaturing lysates. As shown by Figure 4B,C, the BlastR lysis system also allows for reciprocal IP, which is important for validating findings.

### Effective tool for studying low-abundance proteins

It is well established that most PTMs are transient and substoichiometric relative to their parental protein, which makes identification more challenging; particularly when studying signal transduction pathways where PTM changes are often labile [50,51]. Having tools that can capture these modest, but significant, changes are paramount toward understanding the PTMs’ role in the cell. In the present study, three target proteins that ranged from very high (4.18 × 10^8^ molecules/A431 cell; EGFR), medium (4.30 × 10^7^ molecules/A431 cell; Ras), and low in abundance (1.35 × 10^4^ molecules/A431 cell; c-Fos), based on densitometric analysis of cellular content relative to recombinant protein (Supplementary Figure S4), were investigated. The four PTMs that were investigated also varied significantly in signal for each of the three target proteins (Figure 3, and Supplementary Figures S2 and S3), but the Signal-Seeker tools were effective at capturing even the very low abundance modifications, for example c-Fos Ac. Utilizing the Signal-Seeker kits will allow users to isolate and identify a wide dynamic range of PTMs including lower abundance PTM profiles.

### Beneficial tool for studying low-level endogenous and dynamic changes in PTMs

In many cases, novel protein modifications are commonly studied using overexpression and mutagenesis models, which are critical tools to determine how and where a protein is being modified, but can produce erroneous results when used to study physiologic changes. This point was alluded to by Jura et al. [42] in their study of Ras Ub. Data in the present study (Figure 7B) provided compelling evidence that the Signal-Seeker tools can effectively identify dynamic, endogenous changes in PTMs of Ras Ub. Of note, the Ras antibody used in the present study was a pan Ras antibody, and several recent studies have identified that K-Ras, N-Ras, and H-Ras can all be ubiquitinated [52–55]; it is therefore possible that the dynamics of ubiquitinated Ras identified in the present study may represent K-Ras or N-Ras, and not H-Ras, which was the focus of Jura et al. [42] study. This question could be addressed using Signal-Seeker tools in conjunction with Ras isotype-specific antibodies.

These aforementioned studies on Ras Ub [42,52–55] determined that the mono- and di-Ub of Ras affects Ras GTP activity, localization, and downstream kinase signaling; although, there is some disagreement on whether Ras Ub positively or negatively affects Ras activity. Two studies identified di-Ub of Ras as a mechanism to enhance the levels of GTP-activated Ras leading to up-regulation of downstream signaling [52,53], while other studies have shown that Ub of Ras leads to endosomal localization and suppression of downstream signaling [42,55,56]. Researchers from these studies suggest that these differences may arise from isotype and site-specific Ub differences. However, none of these studies investigated the endogenous, dynamic regulation of Ras Ub, which may provide insight into its effect on Ras activity when compared over a timecourse with Ras activation assays and downstream signaling markers. The ability of Signal-Seeker tools to study endogenous, dynamic changes of PTMs make it a very useful tool for mechanistic studies.

## Supporting information

**Supplementary Fig 1.** ***Comparison of BlastR lysis buffer to alternative lysis buffers.*** A431 cells were lysed with BlastR, RIPA, mPER, IP lysis, Denaturing (1% SDS), and Laemmli lysis buffers. Isolation of proteins from the membrane, cytoplasmic, mitochondrial, and nuclear markers were determined using the respective compartment marker proteins.

**Supplementary Fig 2.** ***Detect endogenous changes of all four PTMs for Ras.*** (A). Serum-restricted A431 cells were stimulated with EGF for the given time period. WCL was analyzed for Ras levels. Tubulin was used as a loading control. Unstimulated and EGF treated A431 lysates were incubated with (B) APY03-beads to immunoprecipitate tyrosine-phosphorylated proteins and analyzed for tyrosine-phosphorylated Ras, (C) ASM24-beads to immunoprecipitate SUMOylated 2/3 proteins and analyzed for SUMOylated 2/3 Ras, (D) UBA01-beads to capture ubiquitinated proteins and analyzed for ubiquitinated Ras, (E) and acetyl lysine binding beads to immunoprecipitate acetylated proteins and analyzed for acetylated Ras; shown are representative westerns from N≧3 independent experiments.

**Supplementary Fig 3.** ***Detection of all four PTMs for c-Fos.*** Serum-restricted A431 cells were either unstimulated or stimulated with EGF for 60 minutes prior to lysis with BlastR lysis buffer. Whole cell lysate (WCL) was analyzed for c-Fos levels (lanes 1,2). Ubiquitin control beads (CUB02) were used to immunoprecipitate non-specific binding proteins (lanes 3,4). Ubiquitin binding beads (UBA01) were used to immunoprecipitate ubiquitinated proteins (lanes 5,6). Acetyl lysine binding beads (15E12) were used to immunoprecipitate acetylated proteins (lanes 7,8). Phospho-tyrosine binding beads (APY03) were used to immunoprecipitate tyrosine-phosphorylated proteins (lanes 9,10). SUMO 2/3 binding beads (ASM24) were used to immunoprecipitate SUMOylated 2/3 proteins (lanes 11, 12). IgG binding control beads were used to immunoprecipitate non-specific binding proteins (lanes 13,14). All samples were separated by SDS-PAGE and analyzed by western immunoblotting using a c-Fos antibody to identify changes in c-Fos PTMs in response to EGF. Shown is a representative western from N≧3 independent experiments.

**Supplementary Fig 4.** ***Protein abundance of EGFR, Ras, and c-Fos in A431 cells.*** A431 cells were lysed with BlastR lysis buffer. Sample lysate, as well as EGFR, Ras, and c-Fos recombinant proteins were separated by SDS-PAGE. Samples were analyzed by western blot using EGFR, Ras, and c-Fos antibodies. Densitometic analysis of recombinant EGFR protein was used to establish a standard curve, and concentration of EGFR in A431 cells was determined based on normalization to that standard curve. Ras and c-Fos concentrations were determined using the same method with their respective standard curves.

## Abbreviations

Ac: acetylation
DMEM: Dulbecco’s modified Eagle’s medium
EGF: epidermal growth factor
EGFR: epidermal growth factor receptor
HRP: horseradish peroxidase
IP: immunoprecipitation
NEM: N-Ethylmaleimide
OD: optical density
PTM: post-translational modification
POI: protein of interest
pY: tyrosine phosphorylation
RIPA: radioimmunoprecipitation assay
RT: room temperature
SUMO 2/3: small ubiquitin-like modifier 2/3
TSA: trichostatin A
Ub: ubiquitination
UBD: ubiquitin-binding domain
