# Supplementary material for: A simple toolset to identify endogenous post-translational modifications for a target protein: a snapshot of the EGFR signaling pathway
Source: Biosci Rep. 2017 Aug 2;37(4):BSR20170919. doi: 10.1042/BSR20170919 (PMC6192658; doi:10.1042/BSR20170919)
Supplement: Supplementary file 1 [file bsr-37-bsr20170919-s1.pdf]

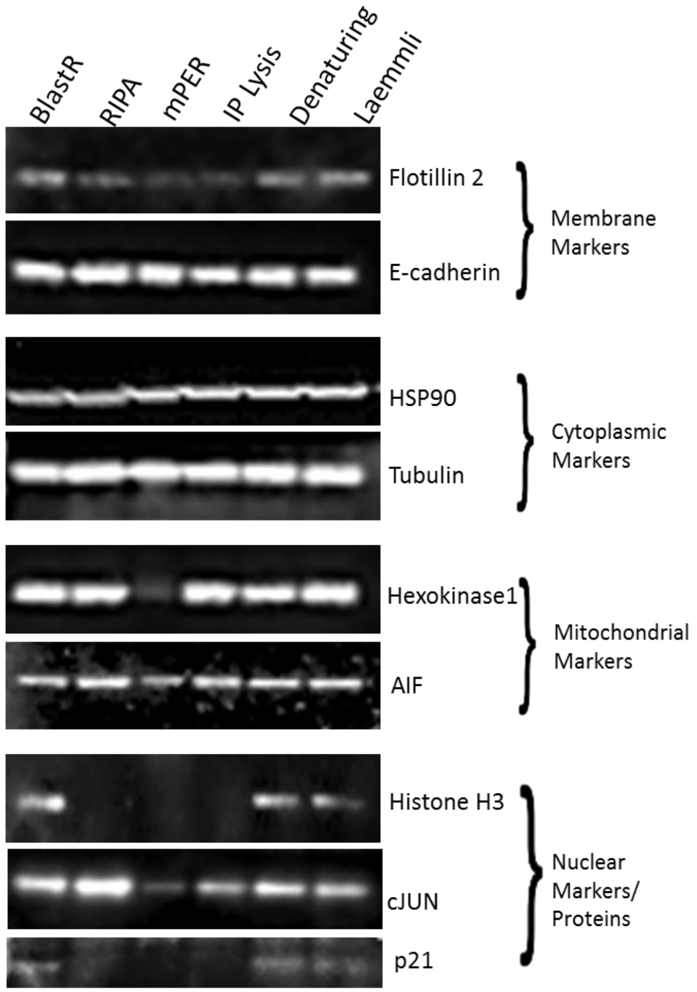

Supplementary Fig 1. Comparison of BlastR lysis buffer to alternative lysis buffers. A431 cells were lysed with BlastR, RIPA, mPER, IP lysis, Denaturing (1% SDS), and Laemmli lysis buffers. Isolation of proteins from the membrane, cytoplasmic, mitochondrial, and nuclear markers were determined using the respective compartment marker proteins.

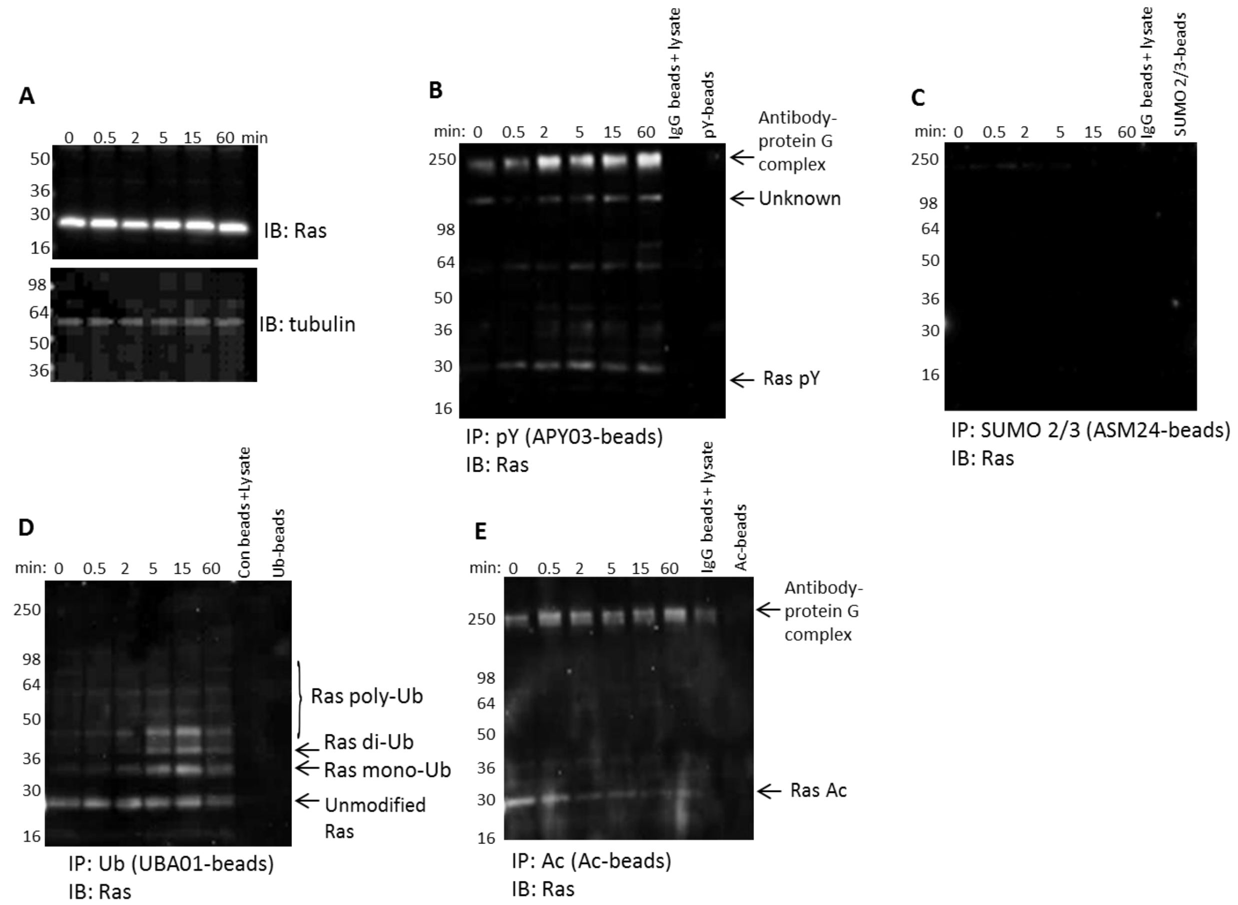

Supplementary Fig 2. Detect endogenous changes of all four PTMs for Ras. (A). Serum-restricted A431 cells were stimulated with EGF for the given time period. WCL was analyzed for Ras levels. Tubulin was used as a loading control. Unstimulated and EGF treated A431 lysates were incubated with (B) APY03-beads to immunoprecipitate tyrosine-phosphorylated proteins and analyzed for tyrosine-phosphorylated Ras, (C) ASM24-beads to immunoprecipitate SUMOylated 2/3 proteins and analyzed for SUMOylated 2/3 Ras, (D) UBA01-beads to capture ubiquitinated proteins and analyzed for ubiquitinated Ras, (E) and acetyl lysine binding beads to immunoprecipitate acetylated proteins and analyzed for acetylated Ras; shown are representative westerns from N≥3 independent experiments.

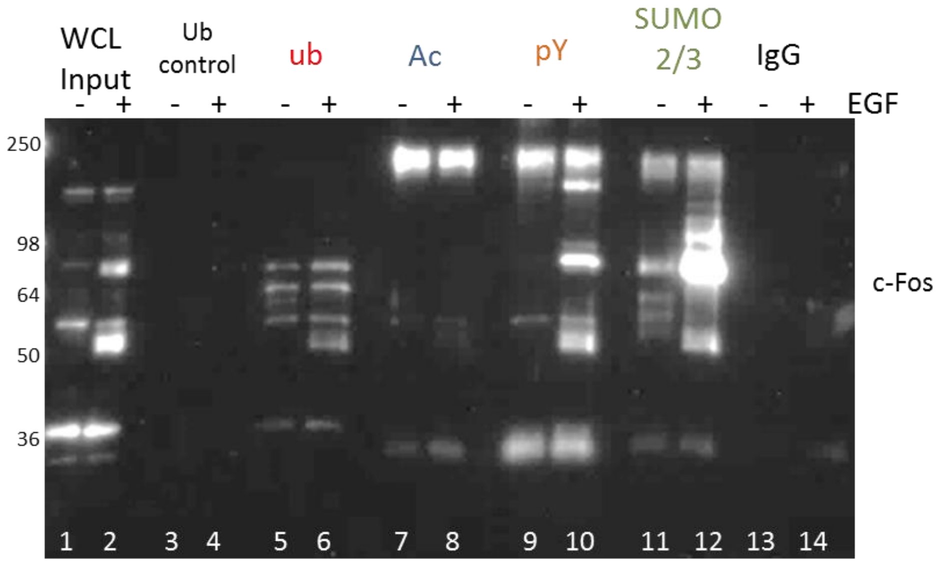

Supplemental Fig 3. Detection of all four PTMs for c-Fos. Serum-restricted A431 cells were either unstimulated or stimulated with EGF for 60 minutes prior to lysis with BlastR lysis buffer. Whole cell lysate (WCL) was analyzed for c-Fos levels (lanes 1,2). Ubiquitin control beads (CUB02) were used to immunoprecipitate non-specific binding proteins (lanes 3,4). Ubiquitin binding beads (UBA01) were used to immunoprecipitate ubiquitinated proteins (lanes 5,6). Acetyl lysine binding beads (15E12) were used to immunoprecipitate acetylated proteins (lanes 7,8). Phospho-tyrosine binding beads (APY03) were used to immunoprecipitate tyrosine-phosphorylated proteins (lanes 9,10). SUMO 2/3 binding beads (ASM24) were used to immunoprecipitate SUMOylated 2/3 proteins (lanes 11, 12). IgG binding control beads were used to immunoprecipitate non-specific binding proteins (lanes 13,14). All samples were separated by SDS-PAGE and analyzed by western immunoblotting using a c-Fos antibody to identify changes in c-Fos PTMs in response to EGF. Shown is a representative western from N≥3 independent experiments.

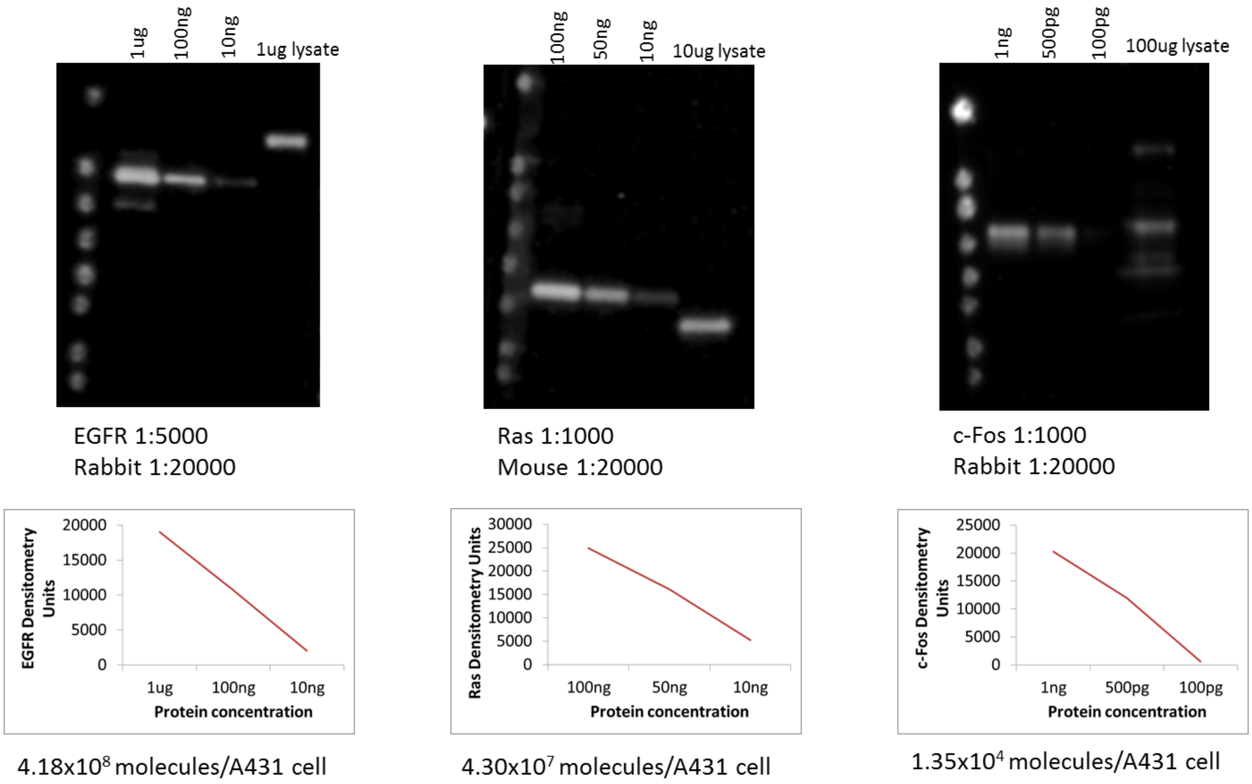

Supplementary Fig 4. Protein abundance of EGFR, Ras, and c-Fos in A431 cells. A431 cells were lysed with BlastR lysis buffer. Sample lysate, as well as EGFR, Ras, and c-Fos recombinant proteins were separated by SDS-PAGE. Samples were analyzed by western blot using EGFR, Ras, and c-Fos antibodies. Densitometic analysis of recombinant EGFR protein was used to establish a standard curve, and concentration of EGFR in A431 cells was determined based on normalization to that standard curve. Ras and c-Fos concentrations were determined using the same method with their respective standard curves.
